# Machine learning reveals sex differences in clinical features of acute exacerbation of chronic obstructive pulmonary disease: A multicenter cross-sectional study

**DOI:** 10.3389/fmed.2023.1105854

**Published:** 2023-03-28

**Authors:** Zhihong Chen, Jiajia Wang, Hanchao Wang, Yu Yao, Huojin Deng, Junnan Peng, Xinglong Li, Zhongruo Wang, Xingru Chen, Wei Xiong, Qin Wang, Tao Zhu

**Affiliations:** ^1^Respiratory Medicine and Critical Care Medicine, Zhongshan Hospital of Fudan University, Shanghai, China; ^2^Rheumatology Medicine, Second Affiliated Hospital of Chongqing Medical University, Chongqing, China; ^3^Respiratory Medicine and Critical Care Medicine, and Preclinical Research Center, Suining Central Hospital, Suining, China; ^4^Respiratory Medicine and Critical Care Medicine, ZhuJiang Hospital of Southern Medical University, Guangzhou, China; ^5^Respiratory Medicine and Critical Care Medicine, Second Affiliated Hospital of Chongqing Medical University, Chongqing, China; ^6^Department of Mathematics, University of California, Davis, CA, United States

**Keywords:** acute exacerbation of chronic obstructive pulmonary disease, sex, binary logistic regression, nomogram, machine learning, K-prototypes algorithm, random forest model, XGBoost model

## Abstract

**Introduction:**

Intrinsically, chronic obstructive pulmonary disease (COPD) is a highly heterogonous disease. Several sex differences in COPD, such as risk factors and prevalence, were identified. However, sex differences in clinical features of acute exacerbation chronic obstructive pulmonary disease (AECOPD) were not well explored. Machine learning showed a promising role in medical practice, including diagnosis prediction and classification. Then, sex differences in clinical manifestations of AECOPD were explored by machine learning approaches in this study.

**Methods:**

In this cross-sectional study, 278 male patients and 81 female patients hospitalized with AECOPD were included. Baseline characteristics, clinical symptoms, and laboratory parameters were analyzed. The K-prototype algorithm was used to explore the degree of sex differences. Binary logistic regression, random forest, and XGBoost models were performed to identify sex-associated clinical manifestations in AECOPD. Nomogram and its associated curves were established to visualize and validate binary logistic regression.

**Results:**

The predictive accuracy of sex was 83.930% using the k-prototype algorithm. Binary logistic regression revealed that eight variables were independently associated with sex in AECOPD, which was visualized by using a nomogram. The AUC of the ROC curve was 0.945. The DCA curve showed that the nomogram had more clinical benefits, with thresholds from 0.02 to 0.99. The top 15 sex-associated important variables were identified by random forest and XGBoost, respectively. Subsequently, seven clinical features, including smoking, biomass fuel exposure, GOLD stages, PaO_2_, serum potassium, serum calcium, and blood urea nitrogen (BUN), were concurrently identified by three models. However, CAD was not identified by machine learning models.

**Conclusions:**

Overall, our results support that the clinical features differ markedly by sex in AECOPD. Male patients presented worse lung function and oxygenation, less biomass fuel exposure, more smoking, renal dysfunction, and hyperkalemia than female patients with AECOPD. Furthermore, our results also suggest that machine learning is a promising and powerful tool in clinical decision-making.

## 1. Introduction

Chronic obstructive pulmonary disease (COPD) is a highly heterogonous disease (1, 2). Congenital biological differences between sexes further intensify this diversity in a variety of aspects of COPD (3, 4). A recent study showed that the prevalence of COPD in male patients (11.9%) was two times as high as that in female patients (5.4%) in China (5). In developing countries, such as China, India, and most of the African countries, the most common risk factor for COPD in women is biomass fuel exposure, whereas smoking is the major risk factor for men (6, 7). It is found that female patients were more vulnerable than male patients to smoking-induced lung function impairments (8). For comorbidities, some studies reported that female patients with COPD were more likely to have anxiety, depression, osteoporosis, inflammatory bowel disease (IBD), chronic heart failure (CHF), and diabetes mellitus (DM) than male patients (9–12). By contrast, alcoholism and ischemic heart disease (IHD) were lower in female patients with COPD (13). The phenotypes of COPD are sex-dependent. It is reported that the proportion of whole-lung emphysema in male patients was higher (14, 15). Interestingly, the emphysema distribution also differs by sex. Compared with male patients, female patients demonstrated a similar proportion of emphysema in the center of the lung but a lower proportion in the peel (15, 16). Åberg et al. (11) found that more inhaled triple therapy [inhaled corticosteroid (ICS) plus long-acting β2 agonist (LABA) plus long-acting muscarinic antagonist (LAMA), ICS/LABA/LAMA], smoking cessation support, and pneumococcal vaccination were administrated, and more sick leaves were taken by female patients with COPD in Sweden. Celli et al. (17) showed that the time to first exacerbation was shorter and the exacerbation rate was 25% higher in female patients in a worldwide multicenter study.

Although a variety of diversities were identified between male and female patients with COPD, the differences in clinical features were rarely investigated in acute exacerbation chronic obstructive pulmonary disease (AECOPD). Meanwhile, we observed many sex-related differences in daily clinical practice. Therefore, the purpose of our study was to answer an interesting question of whether the clinical presentations differ by sex in patients with AECOPD. Furthermore, the degree of sex differences and the sex-associated clinical features in patients with AECOPD were explored. In the present study, the k-prototype algorithm was included to compute the sex differences in patients with AECOPD. Binary logistic regression and machine learning methods, including the XGBoost model and random forest model, were used to identify sex-associated clinical features in patients with AECOPD. The nomogram and its associated curves were built to visualize and verify the binary logistic regression model. The results of this study will inform individualized treatment and evaluate the prognostics in different groups of COPD, and also solve the doubts in our minds.

## 2. Materials and methods

### 2.1. Study design and sites

This multicenter cross-sectional study was carried out in the respiratory departments of the Second Affiliated Hospital of Chongqing Medical University, Zhongshan Hospital of Fudan University, and Zhujiang Hospital of Southern Medical University, from January 2017 to June 2019. The study protocol was approved by the Research Ethics Committee of the Second Affiliated Hospital of Chongqing Medical University (No. 2019–23) and was conducted according to the Declaration of Helsinki. Informed consent was obtained from all the patients by the responsible physician or an appropriately trained staff member. Meanwhile, the past history of all patients with COPD was also reviewed by the responsible physician. Standard care and treatments were provided in our study according to current clinical guidelines (18–20).

### 2.2. Definitions

According to the Global Initiative for Chronic Obstructive Lung Disease (GOLD) (20), a COPD diagnosis was established by a pulmonologist based on a history of exposure to risk factors, including smoking and biomass fuel exposure, clinical presentations, and airflow obstruction measured by spirometry (a post-bronchodilator fixed ratio of FEV1/FVC < 0.7). AECOPD referred to an event characterized by acute changes in clinical symptoms beyond normal day-to-day variation according to the criteria in the GOLD guidelines (20). Meanwhile, due to safety concerns and cooperation of the spirometer test, the lung function test was not performed in AECOPD patients with respiratory failure. Ex-smoker was defined as quitting smoking more than 6 months before data collection (21, 22). Biomass fuel exposure was defined as using an indoor open fire with coal, coke, wood, pellet, agricultural residue, or animal dung at home as a primary means of cooking or heating for ≥ 1 year (23, 24). Neutrophils-to-lymphocytes ratio (NLR) was defined as neutrophils divided by lymphocytes in the blood (25).

### 2.3. Sample size determinations

We determined the size of the study population based on the prevalence of COPD in different sex in China. The prevalence was 11.9% in male patients and 5.4% in female patients (5). Therefore, a minimum total of 224 patients (149 male patients and 75 female patients) were required to detect at least a 40% difference in effect size for an 80% power, assuming *α* = 0.05, and allocation ratio = 2 (21, 25). Furthermore, 20% more (179 male patients and 90 female patients) were included.

### 2.4. Inclusion and exclusion criteria

**Figure 1:**
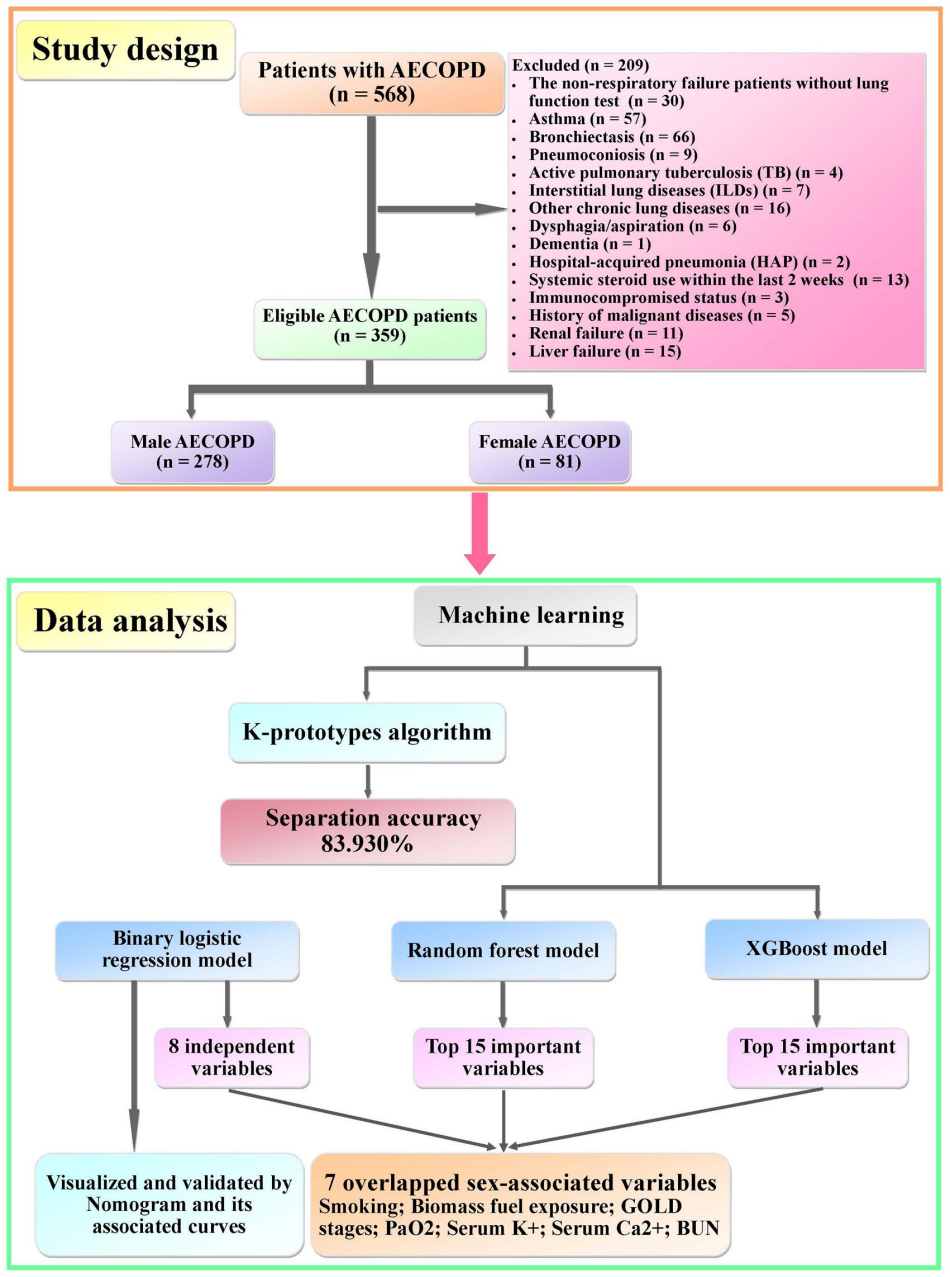
Summary of study design and data analysis.

According to our previous studies (21, 25, 26), inclusion and exclusion criteria were made. The inclusion criterion was patients with COPD exacerbation requiring hospitalization with an age ≥ 40 years old. Exclusion criteria were as follows: non-respiratory failure patients without lung function test, asthma, bronchiectasis, pneumoconiosis, active pulmonary tuberculosis (TB), interstitial lung diseases (ILDs), other chronic lung diseases, dysphagia and aspiration, dementia, hospital-acquired pneumonia (HAP), immunocompromised status (organ transplants, immunosuppressive agents use within the last 4 weeks, and HIV infection), systemic steroid use within the last 2 weeks, history of malignant diseases, renal failure, and liver failure. A total of 568 hospitalized patients with AECOPD were enrolled. Finally, 278 male patients and 81 female patients were eligible and included (Figure 1).

### 2.5. Data collection

Demographics, underlying diseases, comorbid conditions, symptoms, laboratory data, and lung function tests were recorded and collected. The blood samples for laboratory tests and lung function tests were all collected and analyzed within 24 h after admission. All patients underwent chest high-resolution CT (HRCT) scan within 48 h after admission. Meanwhile, the radiation dose of HRCT was in the safe range (27, 28).

### 2.6. Data analysis

Statistical analyses were carried out by SPSS 22.0. The baseline characteristics of the study population were described. According to our previous studies, the data were analyzed (21, 25, 26, 29, 30). In brief, mean ± standard deviation (SD) was used to present normally distributed data. Median and interquartile ranges (IQR) were performed to present non-normally distributed data. Categorical data were expressed as frequencies. Chi-square was used to analyze categorical variables. Continuous variables with normal distribution were analyzed by the Student *t-*test. Ordinal variables and continuous variables without normal distribution were analyzed by the Mann–Whitney *U*-test. The data distribution was examined by the Kolmogorov–Smirnov test. Variables with significant differences (*p* < 0.05) associated with sex by univariate analysis were included in the binary logistic regression model. The Spearman rank method was performed to analyze correlations. A threshold of *p* < 0.05 was considered to be significant. In addition, the nomogram, calibration curve, ROC curve, and DCA curve were established by R version 4.1.2 (22).

### 2.7. Machine learning

In order to explore the degree of sex difference in clinical data, an unsupervised learning procedure was used in this study. In brief, the label of the sex of each patient was taken off. Then, the k-prototype algorithm (Python “kmodes” package) (31, 32), which is a modified k-means algorithm and can handle both continuous and categorical data, was used to cluster the patients and predict the sex. Subsequently, to verify and validate the result of the binary logistic regression model, tree-based models, random forest model (Python “sklearn ensemble” package) (33, 34), and XGBoost model (Python “XGBoost” package) (35, 36) were used to explore sex-associated variables in patients with AECOPD. Then, the top 15 important variables of each model were identified.

## 3. Results

### 3.1. Baseline characteristics of patients with AECOPD

In this study, a total of 568 hospitalized patients with AECOPD were enrolled. In the end, 278 male patients (77.44%) and 81 female patients (22.56%) were eligible (Figure 1). The ratio of male patients to female patients was 3.43. The demographic data of the two groups separated by sex are shown in Table 1. Younger age, lower body mass index (BMI), more smoking, less biomass fuel exposure, more severe GOLD stages, higher rate of coronary artery disease (CAD), and a lower rate of type 2 diabetes (T2DM) were observed in male patients.

**Table 1:** Demographic data of patients with acute exacerbation chronic obstructive pulmonary disease (AECOPD; *n* = 359).

	Male (*n* = 278)	Female (*n* = 81)	Statistical values	*p*
Age (years)	69.7374 ± 8.97084	73.9506 ± 8.28991	−3.782	0.000
Body mass index (BMI)	21.9335 ± 3.55639	23.6837 ± 3.39966	−3.936	0.000
Biomass fuel exposure	20	36	66.108	0.000
*Smoking*			−10.538	0.000
Non-smoking	57	75		
Ex-smoking	81	2		
Current-smoking	140	4		
*GOLD stages*			−3.007	0.003
Stage I: mild (≥ 80%)	30	18		
Stage II: moderate (50–79%)	76	34		
Stage III: severe (30–49%)	81	8		
Stage IV: very severe (< 30%) without respiratory failure	30	2		
Respiratory failure	61	19		
*Underlying diseases/co-morbidities*				
Pleural effusion	9	3	0.042	0.837
Community-acquired pneumonia (CAP)	122	33	0.253	0.615
Coronary artery disease (CAD)	84	15	4.297	0.038
Hypertension	99	38	3.395	0.065
Type 2 diabetes (T2DM)	39	19	4.116	0.042
Atrial fibrillation (Af)	7	4	1.237	0.266
Connective tissue disease (CTD)	2	2	1.743	0.187
Pneumothorax	1	0	0.292	0.589
*Mechanical Ventilation (MV)*			−1.195	0.232
Non-ventilation	253	70		
Non-invasive positive pressure ventilation (NIPPV)	24	11		
Invasive positive pressure ventilation (IPPV)	1	0		

### 3.2. Clinical features of patients with AECOPD

As shown in Table 2, male patients had lower lymphocytes%, PaO_2_, serum calcium (Ca^2+^) and albumin (ALB), and higher serum potassium (K^+^), blood urea nitrogen (BUN), and serum creatinine (Cr), compared with female patients with AECOPD.

**Table 2:** Clinical features and laboratory data of patients with AECOPD (*n* = 359).

	Male (*n* = 278)	Female (*n* = 81)	Statistical values	*p*
Fever	34	5	2.377	0.123
White blood cells (WBC; × 10^9^/L)	8.0017 ± 3.25730	7.7812 ± 3.01039	0.545	0.586
Neutrophils (NS; ×10^9^/L)	5.7995 ± 2.98370	5.5394 ± 2.75333	0.702	0.483
Lymphocytes (LYM; ×10^9^/L)	1.4127 ± 0.71034	1.5746 ± 0.80183	−1.752	0.081
Eosinophils (EOS; ×10^9^/L)	0.2541 ± 0.47394	0.2052 ± 0.19558	0.905	0.366
NS %	70.5810 ± 11.70601	69.2781 ± 11.34658	0.887	0.375
LYM %	19.2803 ± 8.66248	21.7710 ± 9.50319	−2.227	0.027
EOS %	3.2677 ± 3.98462	2.9698 ± 2.97632	0.624	0.533
Neutrophils-to-lymphocytes ratio (NLR)	5.2443 ± 4.98012	5.0769 ± 7.46931	0.235	0.814
Procalcitonin (PCT; ng/ml)	0.1520 ± 0.63657	0.0961 ± 0.25855	0.772	0.441
C-reaction protein (CRP; mg/ml)	23.3460 ± 38.21165	15.7633 ± 21.36604	1.709	0.088
Erythrocyte sedimentation rate (ESR; mm/first hour)	21.1187 ± 19.29751	20.8642 ± 17.42466	0.107	0.915
Air blood gas (ABG)				
PH	7.4142 ± 0.24726	7.4446 ± 0.05010	−1.098	0.273
PaCO2 (mmHg)	43.1432 ± 12.37488	41.2346 ± 9.03088	1.291	0.198
PaO2 (mmHg)^*^	75.0000 ± 21.0000	80.0000 ± 35.5000	−2.190	0.029
Actual base (AB; mmol/L)	27.5176 ± 3.96932	27.6926 ± 4.12216	−0.346	0.729
Standard base (SB; mmol/L)	27.2119 ± 2.65865	27.6099 ± 2.92872	−1.158	0.248
Anion gap (AG)	11.1540 ± 4.81060	11.7237 ± 4.76788	−0.940	0.348
Serum sodium (Na^+^; mmol/L)	138.3791 ± 4.94587	138.0654 ± 5.66417	0.486	0.627
Serum potassium (K^+^; mmol/L)	3.9643 ± 0.42043	3.8448 ± 0.45909	2.204	0.028
Serum calcium (Ca^2+^; mmol/L)^*^	2.2300 ± 0.1800	2.2700 ± 0.1700	−2.748	0.006
Serum magnesium (Mg^2+^; mmol/L)	0.8578 ± 0.09985	0.8358 ± 0.08531	1.804	0.072
Albumin (ALB; g/L)	37.8620 ± 4.21131	39.0765 ± 3.87802	−2.324	0.021
Blood urea nitrogen (BUN) (mmol/L)^*^	6.0750 ± 2.9100	5.3300 ± 1.7400	−2.963	0.003
Creatinine (Cr; μmol/L)^*^	71.3000 ± 22.7300	62.5000 ± 26.1500	−3.515	0.000
Alanine aminotransferase (ALT; U/L)	21.9496 ± 22.13230	20.4815 ± 20.37591	0.535	0.593
Aspartate aminotransferase (AST; U/L)	23.4388 ± 19.15700	23.2346 ± 18.13441	0.085	0.932
Total bilirubin (TBIL; μmol/L)	10.5773 ± 4.86510	10.7160 ± 4.29166	−0.232	0.817
Indirect bilirubin (IBIL; μmol/L)	6.0702 ± 3.04234	6.5457 ± 2.81096	−1.259	0.209
Direct bilirubin (DBIL; μmol/L)	4.5072 ± 2.40947	4.1704 ± 2.06219	1.142	0.254
Random blood glucose (RBG; mmol/L)	6.6057 ± 2.29524	7.1493 ± 2.80825	−1.779	0.076

* Data presented as median ± IQR.

### 3.3. The sex differences of clinical features in patients with AECOPD

**Figure 2:**
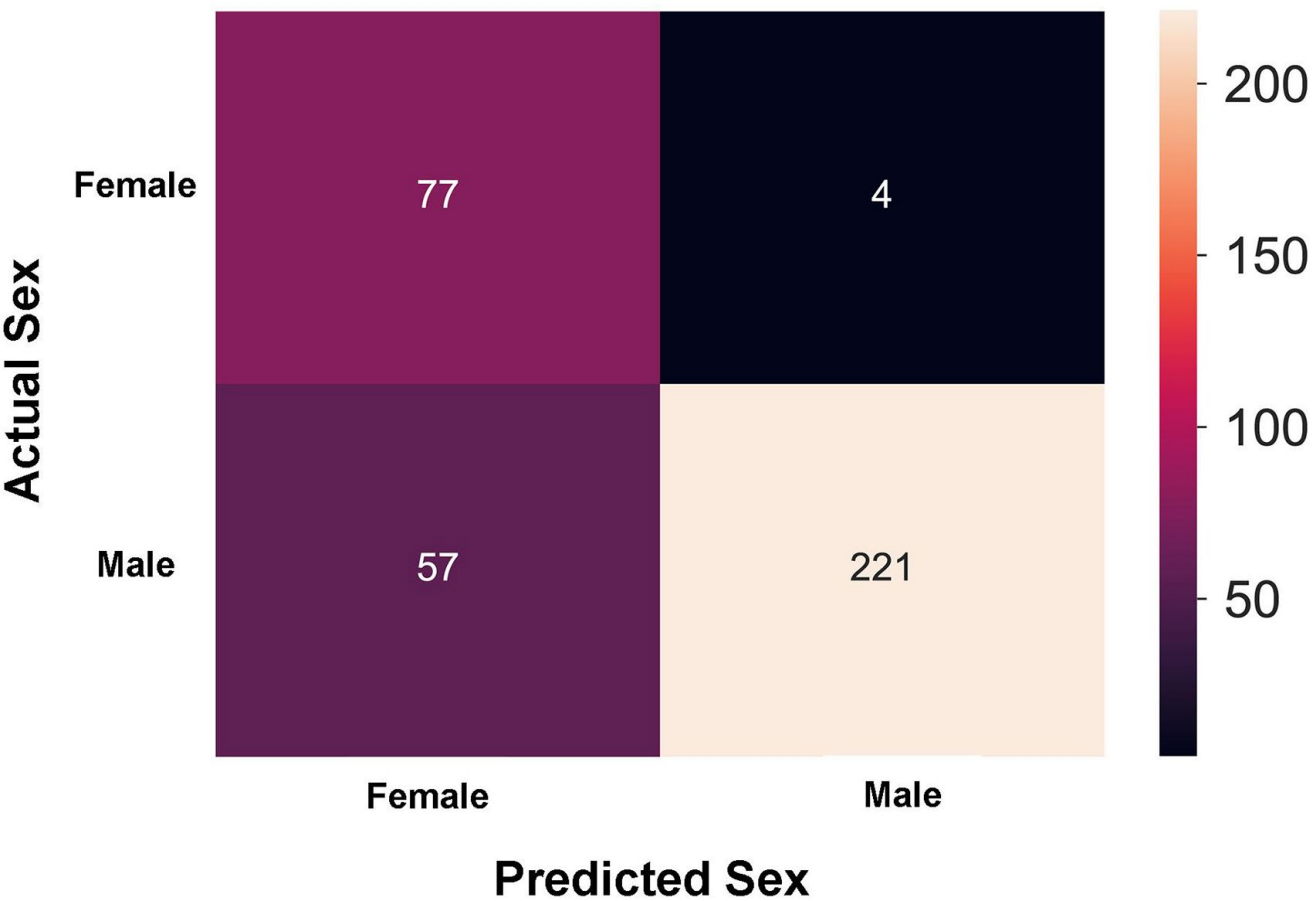
Sex prediction in patients with acute exacerbation chronic obstructive pulmonary disease (AECOPD) by the k-prototype algorithm.

In order to explore whether clinical features could be separated by sex, a k-prototype algorithm was performed. As shown in Figure 2, our predicted labels and true labels were compared using the confusion matrix. The accuracy of separation was 83.930%. A total of 221 out of 278 male patients and 72 out of 76 female patients were predicted correctly.

### 3.4. Binary logistic regression analysis In patients with AECOPD

To explore independent factors associated with sex in hospitalized patients with AECOPD, binary logistic regression was performed. In a binary logistic regression model, 14 factors with significant differences between male patients and female patients in univariate analysis, including age, BMI, smoking, biomass fuel exposure, lung function (GOLD stages), CAD, T2DM, PaO_2_, lymphocytes%, serum K^+^, serum Ca^2+^, ALB, BUN, and serum Cr were included. As shown in Table 3, smoking, biomass fuel exposure, lung function (GOLD stages), CAD, PaO_2_, serum K^+^, and BUN were independently associated with sex in patients with AECOPD. Meanwhile, the value of p of serum Ca^2+^ was at a borderline level of statistical significance (*p* = 0.056).

**Table 3:** Binary logistic regression analysis of independent factors associated with sex in patients with AECOPD (*n* = 359).

	OR	OR 95% C.I.	Sig. (value of *p*)
Smoking			
Ex-smoking	0.025	0.005 ~ 0.134	0.000
Current smoker	0.020	0.005 ~ 0.078	0.000
Biomass fuel exposure	3.456	1.379 ~ 8.664	0.008
GOLD Stages			0.000
Stage II: moderate (50–79%)	1.425	0.451 ~ 4.497	0.546
Stage III: severe (30–49%)	0.233	0.061 ~ 0.885	0.032
Stage IV: very severe (<30%) without respiratory failure	0.045	0.005 ~ 0.370	0.004
Respiratory failure	2.432	0.684 ~ 8.645	0.170
PaO2	1.026	1.011 ~ 1.041	0.001
Serum K^+^	0.316	0.124 ~ 0.804	0.016
Serum Ca^2+^	19.436	0.930 ~ 406.123	0.056
BUN	0.741	0.600 ~ 0.915	0.005
CAD	0.233	0.092 ~ 0.588	0.002
Constant	0.098		0.519

### 3.5. Nomogram was used to visualize and validate the binary logistic regression model

**Figure 3:**
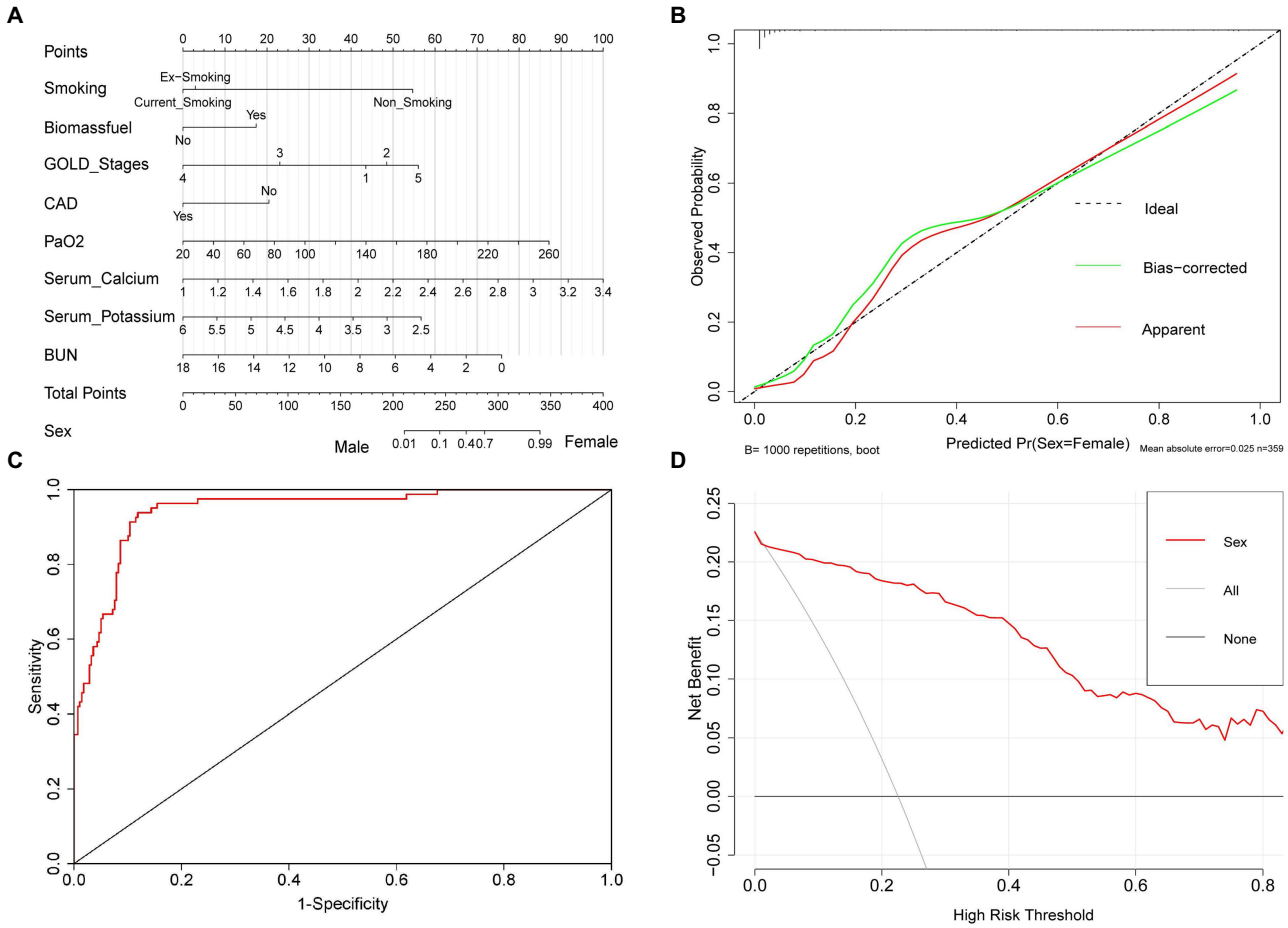
The nomogram for predicting sex in patients with AECOPD. Nomogram was used to visualize and validate the binary logistic regression model. **(A)** Nomogram. The total point of a specific patient is the sum of individual variable points. The predicted probability of sex is on the sex scale, which is corresponding to the total points scale. **(B)** Calibration curve. Ideal line: The nomogram reference line; Apparent line: The actual probability of each patient in our study; Bias-corrected line is adjusted by bootstrap with 1,000 resamples. The length of the vertical lines at the top of the plot represents the number of patients. **(C)** ROC curve. **(D)** DCA curve. None line: the assumption that all patients were men. All line: the assumption that all patients were women. Red line: the nomogram.

Based on our established binary logistic regression model, a nomogram was established. The total point of a specific patient is the sum of individual variable points (Figure 3A). A calibration curve with 1,000 bootstraps presented that both the apparent line and the bias-corrected line were close to the ideal line with mean absolute error (MAE) = 0.026 (Figure 3B). Meanwhile, the AUC of the ROC curve was 0.945 (95% CI: 0.918 ~ 0.972; Figure 3C). In addition, the DCA curve identified that when the threshold ranged from 0.02 to 0.99, using our nomogram to predict the sex probability, yielded more net benefit than the scheme, indicating well clinical applicability of this nomogram (Figure 3D).

### 3.6. Random forest model and XGBoost model were used to compute sex-associated variables in patients with AECOPD

**Figure 4:**
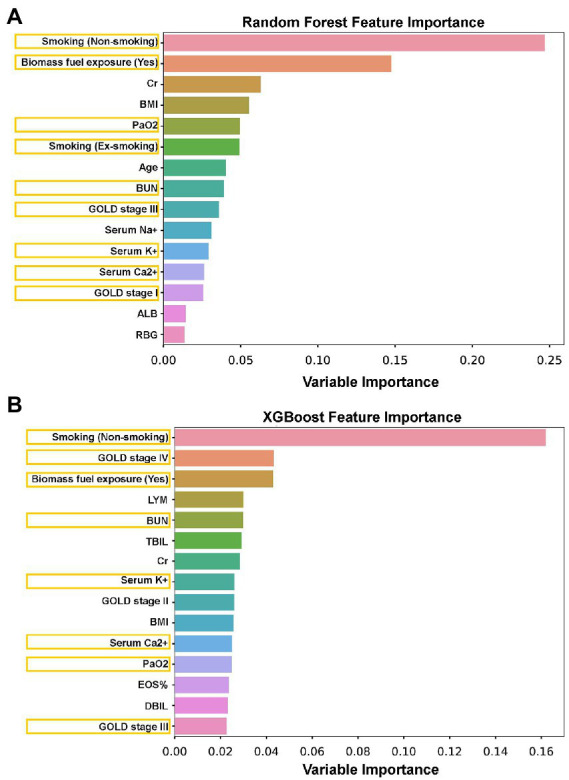
The sex-associated important variables were explored by random forest and XGBoost tree models. **(A)** The top 15 sex-associated important variables were identified by a random forest tree model. **(B)** The top 15 sex-associated important variables were identified by the XGBoost tree model. Orange box: The overlapped variables between the tree model and binary logistic regression.

To further verify and validate the logistic regression model-identified sex-associated clinical variables in patients with AECOPD, both the random forest model and the XGBoost model were performed. In these two machine learning models, the top 15 sex-associated important variables were computed in each model (Figure 4). Compared with the logistic regression model, eight variables [smoking (non-smoking), biomass fuel exposure, PaO2, smoking (ex-smoking), BUN, GOLD stage III, serum K^+^, and serum Ca^2+^] in the random forest model (Figure 4A), and eight variables [smoking (non-smoking), GOLD stage IV, biomass fuel exposure, BUN, serum K^+^, serum Ca^2+^, PaO2, and GOLD stage III] in XGBoost model (Figure 4B) were overlapped, respectively. However, CAD was not found in both two tree models. Furthermore, seven clinical manifestations, including smoking (non-smoking), biomass fuel exposure, PaO2, BUN, GOLD stage III, serum K^+^, and serum Ca^2+^, were overlapped in the logistic regression model, random forest model, and XGBoost model.

### 3.7. Correlations between sex and smoking status, biomass fuel exposure, GOLD stages, CAD, PaO2, serum K^+^, BUN, and serum Ca^2+^ in patients with AECOPD

Since smoking status, biomass fuel exposure, GOLD stages, CAD, PaO_2_, serum K^+^, serum Ca^2+^, and BUN were independently associated with sex in patients with AECOPD by the logistic regression model, their correlations with sex were analyzed by the Spearman rank method. Female patients were significantly negatively correlated with smoking, GOLD stages, CAD, and BUN, as well as positively correlated with biomass fuel exposure, PaO_2_, and serum Ca^2+^ in patients with AECOPD (Table 4). In addition, serum K^+^ was also negatively correlated with female patients with a strong tendency toward statistical significance (*p* = 0.059).

**Table 4:** The correlations between sex and smoking, biomass fuel exposure, GOLD stages, CAD, PaO2, serum K^+^, BUN, and serum Ca2^+^ in patients with AECOPD (*n* = 359).

	Smoking status	Biomass fuel exposure	GOLD Stages	CAD	PaO_2_	Serum K^+^	BUN	Serum Ca^2+^
Female								
R	−0.557	0.429	−0.159	−0.109	0.116	−0.100	−0.157	0.145
*P*	0.000	0.000	0.003	0.038	0.028	0.059	0.003	0.006

## 4. Discussion

This multicenter cross-sectional study included 359 hospitalized patients with AECOPD, 278 male patients (77.44%), and 81 female patients (22.56%). Based on the included baseline characteristics, clinical presentations, and laboratory parameters, the predictive accuracy of sex was 83.930% in patients with AECOPD by the k-prototype algorithm. Binary logistic regression revealed that smoking status, biomass fuel exposure, lung functions (GOLD stages), the rate of coronary artery disease (CAD), PaO_2_, blood urea nitrogen (BUN), serum potassium (K^+^), and serum calcium (Ca^2+^; with the borderline level of statistical significance, *p* = 0.056) were independently associated with sex in patients with AECOPD. Furthermore, the nomogram and its associated curves were used to visualize and validate our binary logistic regression model. MAE of calibration curve = 0.026 and AUC of ROC curve = 0.945 indicate good fitting and high accuracy of our binary logistic regression model. DCA curve presented that our nomogram to predict the sex probability yielded more net benefit than the scheme if the threshold probability was between 0.02 and 0.99. Subsequently, the top 15 sex-associated important variables were computed by the random forest model and XGBoost model, respectively. Furthermore, seven clinical features, including smoking status, biomass fuel exposure, lung functions (GOLD stages), PaO_2_, serum K^+^, serum Ca^2+^, and BUN were simultaneously identified by the logistic regression model, random forest model, and XGBoost model. However, CAD was not identified by the two machine learning models. In addition, we also found that female patients were significantly negatively correlated with smoking, GOLD stages, the rate of CAD, BUN, and serum K^+^ (on the boundary of significance, *p* = 0.059) and were positively correlated with biomass fuel exposure, PaO_2_, and serum Ca^2+^. Collectively, our results indicate that the clinical features differ markedly by sex in patients with AECOPD. Meanwhile, all models showed a high degree of consistency, which validated and enhanced the accuracy of the results. Specifically, male patients presented more smoking and severe lung function loss. Female patients had more biomass fuel exposure. The possibilities of renal dysfunction and electrolyte disorders in male patients were higher than in female patients with AECOPD. Collectively, these results indicate that male patients are inclined to have more comorbidities and complications in hospitalized AECOPD, which potentially lead to worse prognosis.

Chronic obstructive pulmonary disease is one of the leading causes of death all over the world. According to the GOLD, the prevalence is persistently raising in recent decades (20). Meanwhile, COPD is a chronic disease with high heterogeneity (26). Personalized and precise medicine has been the trend in COPD management (25, 26). The prevalence of COPD in male patients was noticeably higher than in female patients. A meta-analysis found that the global prevalence of COPD was 15.70% (13.80 ~ 18.59%) in men and 9.93% (8.73 ~ 11.13%) in women (37). The prevalence of COPD in male patients was 11.9% and in female patients was 5.4% in China (5). In this study, a total of 568 hospitalized patients with AECOPD were enrolled. In the end, 278 male patients and 81 female patients were eligible. The ratio of male patients to female patients was 3.43, which was consistent with the previous study (5).

Growing evidence showed that sex plays a critical role in COPD. The clinical presentations, pathogenesis, prognosis, and treatment response often differ by sex in COPD (1, 3, 4, 17, 38, 39). It is well-known that the risk factors are quite different between men and women (7). Then, the differences in demographic data, risk factors, lung functions (GOLD stages), and comorbidities and complications between sexes were explored in the present study. We found that compared with female patients, male patients were younger, had lower BMI, had more severe smoking status and less biomass fuel exposure, had higher GOLD stages and rate of CAD, and had a lower rate of T2DM in AECOPD by univariate analysis (Table 1). Our data also showed that sex was not associated with the rates of CAP, pneumothorax, pleural effusion, hypertension, atrial fibrillation (Af), and connective tissue disease (CTD) in patients with AECOPD (Table 1).

In addition, although a variety of diversities, such as inhalation therapy response, emphysema distribution, smoking cessation support, pneumococcal vaccination, and sick leaves, were observed between men and women in COPD, the differences in clinical features of AECOPD, particularly in hospitalized AECOPD, were insufficiently explored (11, 14, 15, 40). In our study, the comprehensive laboratory parameters, including blood routine, arterial blood gases (ABG), inflammatory parameters, electrolytes, liver function, and renal function, were collected and analyzed. Compared to female patients with AECOPD, lower lymphocytes%, PaO_2_, serum Ca^2+^, and ALB, and higher serum K^+^, BUN, and Cr were found in male patients by univariate analysis (Table 2).

Based on these 45 clinical variables included in our study, the k-prototype algorithm was performed to explore the degree of sex difference in patients with AECOPD (Figure 2). Our data showed that the accuracy of separation was 83.930%, which suggests that the clinical features are markedly different between male patients and female patients with AECOPD.

Subsequently, 14 factors with significant differences in univariate analysis were included in the binary logistic regression model. Then, it revealed that smoking status, biomass fuel exposure, GOLD stages, the rate of CAD, PaO_2_, serum K^+^, BUN, and serum Ca^2+^ (with a borderline level of statistical significance, *p* = 0.056) were independently associated with sex in patients with AECOPD. Simultaneously, the nomogram and its associated curves were established to visualize and verify the binary logistic regression model (Figure 3A). The ideal line and apparent line were very close in the calibration curve (MAE = 0.025; Figure 3B). Furthermore, the AUC of the ROC curve was 0.945 (95%CI: 0.918 ~ 0.972; Figure 3C). These results indicate good predictive performance and generalizability of the nomogram, which supports the good fitting and high accuracy and efficacy of the binary logistic regression model. In addition, the DCA curve was used to assess the clinical utility of the nomogram, which revealed that our nomogram had greater predictability than the scheme when the threshold ranged from 0.02 to 0.99 (Figure 3D), also indicating this nomogram has good clinical practical value. Collectively, these results indicate good performance of the nomogram in the prediction of sex probability in patients with AECOPD.

Recently, tree-based models, such as the XGBoost model and random forest model, have been widely used in many machine learning tasks and have shown their excellence in disease classification, diagnosis and prognosis prediction, and risk ranking in the medical system (41). Compared to traditional statistical methods, the advantages of the tree-based model can be summarized in the following three points. First, tree-based models do not need strong assumptions on the data distribution and can also be used in numerical and categorical features, which is more universal than linear models like linear regression and logistics regression. Second, the tree-based model is easier to understand by its better visualization. It does not utilize complicated mathematical tools but still gains high performance, compared to support vector machine (SVM) and logistics regression. At last, variables’ contribution can be easily understood by feature importance, which is more convenient than Akaike information criterion (AIC) and Bayesian Information Criterions (BIC) procedures in linear models. Therefore, in order to strengthen the accuracy of prediction and validate the results of logistic the regression model, two tree-based methods, the random forest model and the XGBoost model, were performed. In this study, the top 15 sex-associated important variables were computed by each model (Figure 4). Subsequently, we found that seven clinical features, including smoking status, biomass fuel exposure, lung function (GOLD stages), PaO_2_, serum K^+^, serum Ca^2+^ (borderline level of statistical significance, *p* = 0.056, in logistic regression), and BUN, were simultaneously identified by three models. Our results indicate that these seven clinical features were highly associated with sex in patients with AECOPD. In addition, other clinical features, including CAD, Cr, BMI, age, serum Na+, albumin (ALB), random blood glucose (RBG), lymphocytes (LYM), eosinophils (EOS)%, total bilirubin (TBIL), and direct bilirubin (DBIL), which were identified by one or two models, need to be explored further.

Smoking is the most common risk factor for male patients, whereas biomass fuel burning is the major risk factor for female patients with COPD in developing countries, including China (6, 7). In this study, we also observed that smoking status was worse in male patients, and the rate of biomass fuel exposure was higher in female patients with AECOPD. Furthermore, both smoking status and biomass fuel exposure were independently associated with sex in patients with AECOPD (Table 3), which was consistent with previous studies in other ethnicities (7, 42). Then, these results suggest that switching the energy of cooking and heating from biomass fuel to natural gas or electricity can potentially reduce the risk of COPD in female patients.

The difference in lung function impairment between men and women in COPD was still under debate. In Greece, a nationwide study screened 6,125 patients with COPD and found that female patients were characterized by milder GOLD stages (12). However, a population-based study in Sweden, with 403 male patients and 333 female patients, reported that the difference in FEV1% was not significant between men and women in patients with stable COPD (9). A prospective observational study in Turkey, with 128 patients with stable COPD (86 men and 42 women), showed no differences in FEV1%, FVC%, FEV1/FVC%, and COPD assessment test (CAT) scores between male patients and female patients (40). In our study, due to safety concerns and cooperation of the spirometer test, the lung function test was not performed in patients with AECOPD with respiratory failure. We found that the GOLD stages were independently associated with sex in patients with AECOPD. The GOLD stages in male patients were significantly higher than in female patients. This result indicates that the lung function impairments in male patients with AECOPD are more severe than in female patients.

In our study, the independent associations between sex and PaO_2_, serum K^+^, serum Ca^2+^ (with a borderline level of statistical significance, *p* = 0.056), and BUN were observed in patients with AECOPD (Table 3; Figure 3A). Among them, PaO_2_ and serum Ca^2+^ were lower, and serum K^+^ and BUN were higher in male patients. We speculated that lower PaO_2_ in male patients was probably caused by more severe lung function loss (higher GOLD stages). Barakat et al. (43) found that the risk of acute kidney injury (AKI) was increased in COPD. The multivariable regression revealed that being male, old age, and lower glomerular filtration rate (GFR) were the predictors of AKI and death in patients with COPD. Meanwhile, Fabbian et al. (44) also showed that an old male patient was an independent predictor of AKI in patients with AECOPD. Consistent with previous studies (43–45), our finding revealed that BUN in male patients was significantly higher than in female patients with AECOPD, which probably led to more electrolyte disorders in male patients, featuring by the significant difference in serum K^+^ and serum Ca^2+^ between two sexes. Furthermore, we also observed the positive and significant correlations between female patients and biomass fuel exposure, PaO_2_, and serum Ca^2+^, as well as negative and significant correlations between female patients and smoking, GOLD stages, CAD, and BUN. Meanwhile, serum K^+^ was negatively correlated with female patients at the brink of significance (*p* = 0.059; Table 4; Figure 3A). Then, these data indicate that male patients were more likely to suffer from respiratory failure and renal dysfunction, subsequently, leading to more electrolyte disorders in patients with AECOPD. Therefore, physicians should beware of renal failure and electrolyte disorders in male patients with AECOPD at the early stage of admission and must treat them in time.

Otherwise, a group of clinicians classified COPD into different phenotypes. Izquierdo-Alonso et al. (46) divided patients with COPD into three clinical phenotypes, emphysema type, chronic bronchitis type, and COPD overlap asthma (ACO) type. They found that patients of emphysema type presented worse pulmonary function and greater dyspnea than other patients. According to the Spanish COPD guideline (GesEPOC), COPD was classified into four phenotypes based on the exacerbation frequency and dominant clinical manifestations, including non-exacerbator with emphysema or chronic bronchitis (Type A), mixed COPD-asthma (Type B), exacerbator with emphysema (Type C), and exacerbator with chronic bronchitis (Type D) (47). Based on GesEPOC, Chai et al. (48) explored the clinical differences among four types of COPD. Their results indicated that exacerbator with chronic bronchitis (Type D) patients present poorer disease-specific HRQoL, higher CAT scores, and St George’s Respiratory Questionnaire for COPD (SGRQ-c) scores than the other three types (48). Thus far, no putative diagnosis criteria are made, which may lead to an inaccurate diagnosis of asthma–COPD overlap (ACO). In the current study, 57 COPD combined with asthma were excluded to reduce the confounders. Nevertheless, the difference between COPD and ACO is a critical area to explore in the future.

In the present study, three models, the binary logistic regression model, random forest model, and XGBoost model, were performed to reveal the sex-associated clinical features in patients with AECOPD, promoting the accuracy of our prediction. Nomograms and their associated curves were used to visualize and verify the binary logistic regression model. These were the major strengths of our study. Meanwhile, relatively comprehensive clinical manifestations and laboratory parameters were recorded and collected, which accurately identified the severity and complications of the underlying diseases. Particularly, a chest HRCT scan was performed on each patient. This was critical to exclude interstitial lung diseases (ILDs), bronchiectasis, lung cancer, pneumoconiosis, and other chronic pulmonary diseases, and to diagnose the co-morbidities and complications of COPD, such as CAP, pneumothorax, and pleural effusion, which were the major confounders in COPD study and hard to identify without chest CT in clinical practice. Due to the cross-sectional design, the differences in the responses and effects of the treatments between men and women were not observed and analyzed. Data on the treatments in the stable phase of COPD also was not included in this study, due to the low-quality data on treatment history. Meanwhile, although 359 patients with AECOPD were included in this study, the ratio of male patients to female patients was 3.43, leading to a comparably small sample size in female patients, which was also the major obstacle to performing subgroup or stratified analysis. Therefore, further studies with larger sample sizes of female patients need to be performed to validate these findings. In addition, our data were from Chinese patients which reduced confounders in the analysis. However, the findings from this study need to be replicated in other ethnicities to make them more generalizable.

## 5. Conclusion

Collectively, our results indicate that the clinical presentations, laboratory parameters, and co-morbidities differ obviously by sex in patients with AECOPD. We revealed that smoking, biomass fuel exposure, GOLD stages, PaO_2_, BUN, serum K^+^, and serum Ca^2+^ were independently associated with sex in patients with AECOPD. Specifically, male patients presented more frequent smoking and less biomass fuel exposure, had higher GOLD stages, lower PaO_2_, and a higher risk of renal dysfunction and electrolyte disorders in AECOPD. Our results suggest that the prevention and disease control strategy of COPD should be sex-dependently implemented. In contrast to quitting smoking is more essential for men in preventing COPD, updating the cooking system and using clean energy can potentially reduce the risk of COPD in women, particularly in developing countries and rural areas. Renal function tests and serum electrolyte panels are recommended in male patients with AECOPD at the early stage of admission. Overall, the clinical features in male patients are more severe than in female patients with AECOPD. In addition, our data also indicate that the machine learning approach is a useful and promising tool in COPD management, which could assist physicians to make better prognoses and risk predictions.

## Data availability statement

The original contributions presented in the study are included in the article/supplementary material, further inquiries can be directed to the corresponding author.

## Ethics statement

The studies involving human participants were reviewed and approved by the Research Ethics Committees of Second Affiliated Hospital of Chongqing Medical University (No. 2019-23). The patients/participants provided their written informed consent to participate in this study.

## Author contributions

TZ conceived the study design. JP, JW, HD, ZC, XL, XC, QW, and WX collected, checked, and collated data. ZW proposed and built machine learning models. HW established the nomogram, the calibration curve, the ROC curve, and the DCA curve. ZC, JW, and TZ performed other data analyses. JW, ZC, TZ, ZW, HW, and YY interpreted the results. ZC, JW, HW, YY, and TZ drafted and revised the manuscript. All authors contributed to the article and approved the submitted version.

## Funding

This study was supported by National Natural Science Foundation of China (81970023), National Natural Science Foundation of China (Youth Program), (8180011074), Shanghai Top-Priority Clinical Key Disciplines Construction Project (2017ZZ02013), Chongqing Health Joint Medical Research Project (2020MSXM112), and Natural Sciences Foundation of Sichuan (23NSFSC0667).

## Conflict of interest

The authors declare that the research was conducted in the absence of any commercial or financial relationships that could be construed as a potential conflict of interest.

## Publisher’s note

All claims expressed in this article are solely those of the authors and do not necessarily represent those of their affiliated organizations, or those of the publisher, the editors and the reviewers. Any product that may be evaluated in this article, or claim that may be made by its manufacturer, is not guaranteed or endorsed by the publisher.

## Abbreviations

AB: actual base
AECOPD: acute exacerbation of chronic obstructive pulmonary disease
Af: atrial fibrillation
AG: anion gap
ALB: albumin
ALT: alanine aminotransferase
AST: aspartate aminotransferase
BMI: body mass index
BUN: blood urea nitrogen
Ca^2+^: serum calcium
CAD: coronary artery disease
CAP: community-acquired pneumonia
CHF: chronic heart failure
COPD: chronic obstructive pulmonary disease
Cr: creatinine
CRP: C-reaction protein
CTD: connective tissue disease
DBIL: direct bilirubin
DM: diabetes mellitus
EOS: eosinophils
ESR: erythrocyte sedimentation rate
FEV1: forced expiratory volume in one second
FVC: forced vital capacity
GOLD: global initiative for chronic obstructive lung disease
HAP: hospital-acquired pneumonia
HIV: human immunodeficiency virus
HRCT: high-resolution CT
IBD: inflammatory bowel disease
IBIL: indirect bilirubin
ICS: inhaled corticosteroid
IHD: ischemic heart disease
ILDs: interstitial lung diseases
IPPV: invasive positive pressure ventilation
IQR: median and interquartile ranges
K^+^: serum potassium
LABA: long-acting β2 agonist
LAMA: long-acting muscarinic antagonist
LYM: lymphocytes
Mg^2+^: serum magnesium
MV: mechanical ventilation
Na^+^: serum sodium
NIPPV: non-invasive positive pressure ventilation
NLR: neutrophils-to-lymphocytes ratio
NS: neutrophils
PaCO_2_: partial pressure of carbon dioxide
PaO_2_: partial pressure of oxygen
PCT: procalcitonin
RBG: random blood glucose
SB: standard base
SD: standard deviation
T2DM: type 2 diabetes
TB: tuberculosis
TBIL: total bilirubin
WBC: white blood cells
